# Mitochondrial Structure and Function in the Metabolic Myopathy Accompanying Patients with Critical Limb Ischemia

**DOI:** 10.3390/cells9030570

**Published:** 2020-02-28

**Authors:** Thomas Groennebaek, Tine Borum Billeskov, Camilla Tvede Schytz, Nichlas Riise Jespersen, Hans Erik Bøtker, Rikke Kathrine Jentoft Olsen, Nikolaj Eldrup, Joachim Nielsen, Jean Farup, Frank Vincenzo de Paoli, Kristian Vissing

**Affiliations:** 1 Department of Public Health, Aarhus University, 8000 Aarhus, Denmark; tg@ph.au.dk (T.G.); camilla.tvedeschytz@yahoo.com (C.T.S.); 2 Department of Biomedicine, Aarhus University, 8000 Aarhus, Denmark; tine@clin.au.dk (T.B.B.); jean@biomed.au.dk (J.F.); 3 Department of Cardiothoracic and Vascular Surgery, Aarhus University Hospital, 8200 Aarhus, Denmark; 4 Department of Sports Science and Clinical Biomechanics, University of Southern Denmark, 5230 Odense, Denmark; jnielsen@health.sdu.dk; 5 Department of Cardiology, Aarhus University Hospital, 8200 Aarhus, Denmark; n.riise.jespersen@clin.au.dk (N.R.J.); heb@dadlnet.dk (H.E.B.); 6 Research Unit for Molecular Medicine, Aarhus University Hospital, 8200 Aarhus, Denmark; rikke.olsen@clin.au.dk; 7 Department Vascular Surgery, Rigshospitalet, Copenhagen University, 2100 Copenhagen, Denmark; nikolaj.eldrup@regionh.dk

**Keywords:** mitochondria, myopathy, peripheral artery disease, ultrastructure, bioenergetics, biomarkers

## Abstract

Mitochondrial dysfunction has been implicated as a central mechanism in the metabolic myopathy accompanying critical limb ischemia (CLI). However, whether mitochondrial dysfunction is directly related to lower extremity ischemia and the structural and molecular mechanisms underpinning mitochondrial dysfunction in CLI patients is not understood. Here, we aimed to study whether mitochondrial dysfunction is a distinctive characteristic of CLI myopathy by assessing mitochondrial respiration in gastrocnemius muscle from 14 CLI patients (65.3 ± 7.8 y) and 15 matched control patients (CON) with a similar comorbidity risk profile and medication regimen but without peripheral ischemia (67.4 ± 7.4 y). Furthermore, we studied potential structural and molecular mechanisms of mitochondrial dysfunction by measuring total, sub-population, and fiber-type-specific mitochondrial volumetric content and cristae density with transmission electron microscopy and by assessing mitophagy and fission/fusion-related protein expression. Finally, we asked whether commonly used biomarkers of mitochondrial content are valid in patients with cardiovascular disease. CLI patients exhibited inferior mitochondrial respiration compared to CON. This respiratory deficit was not related to lower whole-muscle mitochondrial content or cristae density. However, stratification for fiber types revealed ultrastructural mitochondrial alterations in CLI patients compared to CON. CLI patients exhibited an altered expression of mitophagy-related proteins but not fission/fusion-related proteins compared to CON. Citrate synthase, cytochrome c oxidase subunit IV (COXIV), and 3-hydroxyacyl-CoA dehydrogenase (β-HAD) could not predict mitochondrial content. Mitochondrial dysfunction is a distinctive characteristic of CLI myopathy and is not related to altered organelle content or cristae density. Our results link this intrinsic mitochondrial deficit to dysregulation of the mitochondrial quality control system, which has implications for the development of therapeutic strategies.

## 1. Introduction

Lower extremity peripheral artery disease (PAD) is a chronic condition most frequently caused by obstructive atherosclerosis [1]. The most devasting manifestation of PAD is critical limb ischemia (CLI). CLI patients suffer from poor functional capacity, severe muscle wasting, low quality of life, and increased amputation and mortality rates [1,2,3]. Surgical intervention may alleviate local symptoms but does not restore functional capacity, and 30 day readmission rates are greater than 40% [4,5]. This highlights the need for novel therapeutic strategies. CLI patients develop a myopathy in the ischemic muscle tissue that is characterized by chronic metabolic, morphological, and functional changes [6,7,8]. Targeting this myopathy represents a potential strategy to improve clinical outcomes in CLI patients.

Preclinical and observational studies indicate that the myopathy of PAD patients is characterized by mitochondrial dysfunction [9,10,11,12,13,14]. In a recent report, CLI patients were shown to possess a distinct bioenergetic signature than patients with less severe manifestations of PAD [15]. Furthermore, therapeutic targeting of mitochondria has been shown to improve recovery from limb ischemia in a murine model of CLI [16]. Similarly, mitochondria-targeted overexpression of catalase has been shown to alleviate the ischemic myopathy in high-fat-diet-fed mice [17]. As mitochondria play an important role in apoptosis and reactive oxygen species production, this raises the likelihood that mitochondrial dysfunction contributes to development of the myopathy. However, CLI patients often present with a high burden of comorbidities and use a wide array of medication, which are, themselves, inducers of mitochondrial dysfunction [18,19]. To further our understanding of whether mitochondrial dysfunction in CLI patients is directly linked to lower extremity ischemia, it is, therefore, essential to establish a control group with a similar medication regimen and co-morbidity risk profile. Furthermore, to develop effective countermeasures, it is vital to understand the evolution of mitochondrial dysfunction. Specifically, mitochondrial dysfunction can reflect reduced organelle content and/or intrinsic changes in the mitochondrial reticulum such as disruption of cristae morphology, but it is yet unclear whether mitochondrial dysfunction in CLI patients relates to altered organelle content or intrinsic abnormalities.

In the current study, we aimed to clarify if impaired mitochondrial function is directly related to lower extremity ischemia by comparison to a matched control group with a similar medication regimen and comorbidity risk profile but without lower leg ischemia. Furthermore, we asked whether inferior mitochondrial function in CLI patients is linked to altered total, sub-population, or fiber-type-specific mitochondrial volumetric content and/or intrinsic changes in mitochondrial cristae density by utilization of transmission electron microscopy. To get an indication of the molecular mechanisms, we further investigated whether CLI patients are characterized by dysregulated mitophagy and/or fission/fusion-related processes that regulate mitochondrial content and structure. The unique data on mitochondrial volumetric content also allowed us to ask whether commonly used biomarkers of mitochondrial content are valid in patients with cardiovascular disease. We demonstrate that impaired mitochondrial respiratory function is indeed a distinct characteristic of the myopathy accompanying CLI. In contrast to other conditions characterized by mitochondrial dysfunction, the respiratory deficit in CLI muscle is unrelated to mitochondrial content and structure. Instead, our results point toward dysregulation of the mitochondrial quality control system PTEN-induced kinase 1 (PINK1)-Parkin-mediated mitophagy as a potential mechanism for suboptimal mitochondrial function in CLI patients.

## 2. Materials and Methods

### 2.1. Participants

PAD patients with CLI (Fontaine stage 3–4) admitted for surgical revascularization of the lower limb were eligible for inclusion. Diagnosis of PAD was confirmed by medical history, physical examination, computed tomography angiography, and ankle brachial index (ABI) (all had ABI < 0.9). Patients with ischemic heart disease admitted for coronary artery bypass grafting were included as controls (CON). These patients were chosen as the CON group because lifestyle and major risk factors for coronary artery disease resemble those of PAD: diabetes mellitus, dyslipidemia, hypertension, smoking, sedentary lifestyle, and advancing age [1]. Only patients with multi-coronary artery disease, a normal ejection fraction, in whom the saphenous vein was collected and used as graft, and with normal lower extremity blood flow (i.e., ABI between 0.9 and 1.4) and no medical history of PAD symptoms were eligible for inclusion. All patients were anesthetized using rocuronium, sevoflurane, fentanyl, and propofol. For both CLI and CON patients, exclusion criteria comprised age < 50/> 75, active chemo or radiation therapy, thiazolidinedione treatment, polymyalgia, lipodystrophy conditions, and hemodialysis treatment. Patients from both groups were included at random. All participants gave written informed consent prior to inclusion. The study was approved by The Central Denmark Region Committee on Health Research Ethics (1-10-72-115-17) and conformed to the standards for human experimental trials outlined in the Declaration of Helsinki.

### 2.2. Ankle-Brachial Index

Prior to inclusion, ABI was measured in accordance with recommended guidelines from the American Heart Association [20] using a pneumatic cuff (Welch Allyn, Skaneateles Falls, NY, USA) and a doppler probe (Parks Medical Electronics, Aloha, OR, USA). The cuff was inflated 20 mmHg above a pressure corresponding to cessation of the flow signal and then carefully deflated until reappearance of the flow signal. The cuff pressure corresponding to the reappearance of flow was considered equivalent to systolic blood pressure. Ankle pressure measurements were performed using the dorsalis pedis artery. After determination of systolic ankle pressure, brachial systolic blood pressure was determined. The highest systolic blood pressure of that measured in each arm was used for calculation of the ABI. The same investigator performed all ABI measurements.

### 2.3. Muscle Samples

Muscle tissue was collected from CLI patients admitted to revascularization surgery and CON patients suffering from coronary artery disease admitted to coronary artery bypass grafting at the Department of Cardiothoracic and Vascular Surgery, Aarhus University Hospital. In CLI patients, muscle tissue was collected from the medial gastrocnemius muscle of the symptomatic leg during surgery under general anesthesia. In CON patients, muscle tissue was collected from the medial gastrocnemius muscle in relation to harvesting the saphenous vein during coronary artery bypass graft surgery under general anesthesia. All muscle samples were harvested after overnight fasting.

**Figure 1:**
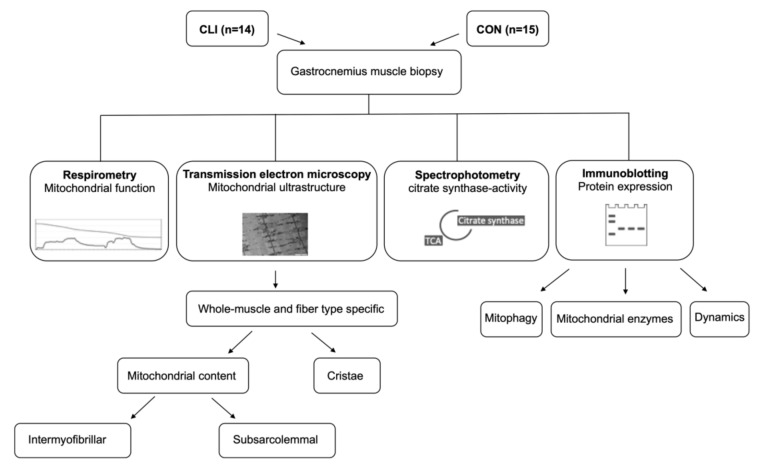
Experimental overview. Muscle biopsies were collected from 14 critical limb ischemia (CLI) patients and 15 control (CON) patients. Mitochondrial respiratory function was analyzed with high-resolution respirometry. Mitochondrial volumetric content and cristae surface area density were analyzed in whole muscle and stratified by fiber type with transmission electron microscopy. The volumetric content stratified by subcellular localization (intermyofibrillar, IMF; subsarcolemmal, SS) was also analyzed. Citrate synthase activity was analyzed with spectrophotometry. Expression of mitophagy-related proteins, fission/fusion-related proteins (dynamics), and mitochondrial enzymes involved in oxidative metabolism were analyzed with immunoblotting.

After removal of visible fat and connective tissue, the muscle sample was separated into four parts (Figure 1). A portion of the sample (~30 mg wet weight) was immediately submerged in an ice-cold relaxing buffer (BIOPS; in mmol L^−1^: 2.77 CaK_2_EGTA, 7.23 EGTA, 20 taurine, 6.56 MgCl_2_, 5.77 ATP, 15 phosphocreatine, 0.5 dithiothreitol, and 50 4-morpholineethanesulphonic acid; pH 7.1) and transferred to the laboratory for analysis of mitochondrial respiratory function. Another part (~1 mm^3^) was fixed for 24 h with 2.5% glutaraldehyde in 0.1 M sodium cacodylate buffer (pH 7.3), rinsed 4 × 15 min in 0.1 M sodium cacodylate buffer, and stored in 0.1 M sodium cacodylate buffer until further processing for transmission electron microscopy analysis. The remaining two samples were snap frozen in liquid nitrogen and stored (−80 °C) until further processing for spectrophotometric and immunoblotting analyses.

### 2.4. Preparation of Permeabilized Muscle Fibers

The muscle sample was transferred to a petri dish filled with BIOPS and placed on ice. Using the tip of two sharp forceps, the muscle sample was carefully dissected into two separate muscle fiber bundles (~2.5 mg wet weight), while the remaining muscle tissue was snap frozen in liquid nitrogen and stored (−80 °C) until further analysis of citrate synthase (CS) activity. Chemical permeabilization of the muscle fiber bundles was achieved by gentle agitation in ice-cold BIOPS buffer containing saponin (50 µg mL^−1^) for 30 min. Following permeabilization, the fiber bundles were rinsed by gentle agitation in an ice-cold respiration medium (MiR05; in mmol L^−1^: 110 sucrose, 60 K-lactobionate, 0.5 EGTA, 0.1% BSA, 3 MgCl_2_, 20 taurine, 10 KH_2_PO_4_, and 20 HEPES; pH 7.1) for 2 × 10 min. Finally, the fibers were blotted dry on filter paper and the wet weight of the muscle fiber bundles was measured using a microbalance (Mettler-Toledo, Greifensee, Switzerland).

### 2.5. High-Resolution Respirometry

Mitochondrial respiratory function was analyzed in duplicate at 37 °C using a two-chamber high-resolution respirometer (Oroboros Instruments, Innsbruck, Austria), as previously described [21,22]. Sequential titrations of substrates and inhibitors were performed in the following order and concentrations: (i) Glutamate (10 mmol L^−1^) and malate (2 mmol L^−1^) to assess state 2 leak respiration; (ii) ADP (5 mmol L^−1^) to assess state 3 respiration with complex I substrates; (iii) cytochrome c (10 μmol L^−1^) to assess the integrity of the outer mitochondrial membrane with an increase in respiration of >10% considered as a sign of damage leading to elimination of data; (iv) succinate (10 mmol L^−1^) to assess state 3 respiration with complex I and II substrates; (v) oligomycin (2 μg mL^−1^) to assess state 4 respiration; (vi) rotenone (0.5 μmol L^−1^) and antimycin A (2.5 mmol L^−1^) to asses residual oxygen consumption. The rate of respiration was expressed relative to mg fiber wet weight, mitochondrial volumetric content, CS activity, and cristae surface area. Data analysis was performed using DatLab software (Oroboros Instruments Innsbruck, Austria) by a blinded investigator.

### 2.6. CS Activity

CS activity was measured with a CS Assay Kit (Cat. CS0720, Sigma-Aldric, St. Louis, MO, USA) as previously described [22]. Briefly, muscle homogenates were diluted 25 times in a reaction mix (Assay Buffer, 30 mM Acetyl CoA solution, 10 mM DTNB solution) and loaded on a 96 well half plate. The 96 well half plate was placed in a spectrophotometer (PHERAstar FS, BMG LABTECH, Ortenberg, Germany) and the background activity at 412 nm was determined. Following background activity measurement, 5 μL oxaloacetate (10 mmol L^−1^) was added to each well to initiate the reaction catalyzed by CS. Light absorbance at 412 nm was measured every 10 s for 5 min at 37 °C. CS activity was calculated from the linear change in absorbance over time and normalized to total protein content, which was measured using a Pierce 660 nm protein assay (Cat. 22660 Thermo Scientific, Rockford, IL, USA).

### 2.7. Transmission Electron Microscopy

**Figure 2:**
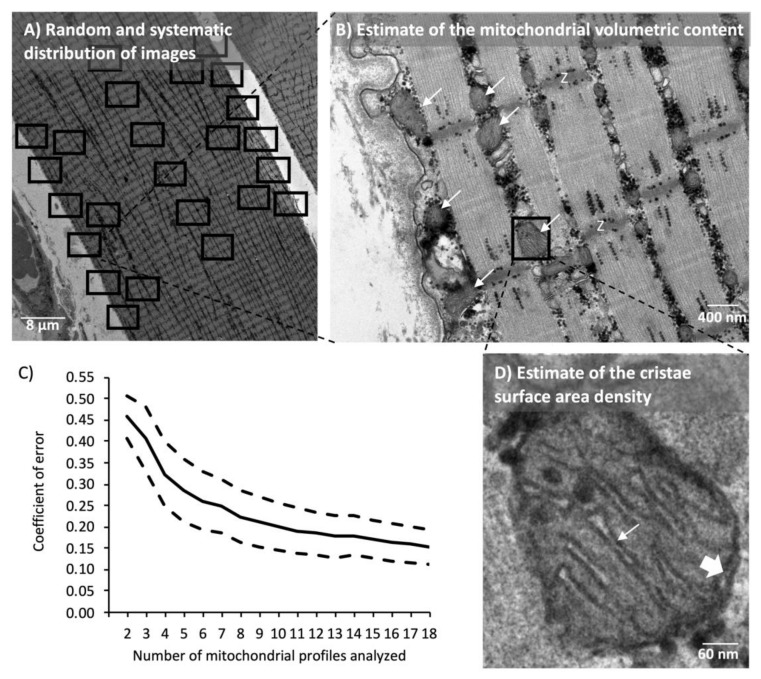
Analysis of mitochondrial volumetric content and cristae surface area density by transmission electron microscopy. (**A**) 24 images were photographed in a randomized systematic order. (**B**) Representative image used for the quantification of the mitochondrial volumetric content. White arrows point at examples of mitochondrial profiles. Z indicates Z-disc. (**C**) Mean (continuous line) and 95% CI (dashed lines) of coefficient of error after analysis of up to 18 profiles (n = 27 subjects). (**D**) Representative mitochondrial profile used for quantification of cristae surface area density. The thin arrow points at one cristae with a double-leaflet. The thick arrow points at an example of the double-leaflet of the outer membrane.

The fixed muscle specimens were postfixed for 90 min at 4 °C with 1% osmium tetroxide (OsO_4_) and 1.5% potassium ferrocyanide (K_4_Fe(CN)_6_) in 0.1 M sodium cacodylate buffer. Afterward, the specimens were rinsed twice in 0.1 M sodium cacodylate buffer at 4 °C, dehydrated through a graded series of alcohol at 4–20 °C, infiltrated with graded mixtures of propylene oxide and Epon at 20 °C, and embedded in 100% Epon at 30 °C. Ultra-thin (60 nm) sections were cut using a Ultracut UCT ultramicrotome (Leica Microsystems, Wetzlar, Germany) in at least three depths separated by 150 μm and contrasted with uranyl acetate and lead citrate. Sections were examined and photographed in a pre-calibrated transmission electron microscope (JEM-1400 Plus, JEOL Ltd., Tokyo, Japan) and a Quemesa camera. All longitudinal fibers (median of 8.5; range from 8 to 17) were photographed with 10,000× magnification in a randomized systematic order including 12 images from the subsarcolemmal region and 12 images from the myofibrillar region hereof 6 from both the superficial and central regions of the myofibrillar space (Figure 2). Fibers were classified as type 1 or 2 based on Z-disc width [23], where the three fibers with the thickest Z-discs were classified as type 1 fibers and vice versa for type II fibers. All intermediate fibers were discarded from the fiber type analyses. One biopsy (CON) was discarded due to poor quality of the muscle fibers.

The volumetric content (VV) of intermyofibrillar (IMF) and subsarcolemmal (SS) mitochondria were estimated by point counting according to the principle of Cavalieri [24] using the formula AA = VV, where AA is the area fraction of mitochondria. The AA was estimated by point counting [24] using a grid size of 180 nm. To take into account the cylindrical shape of the fiber, the superficial images of the myofibrillar space were weighted three times higher than the central images. IMF mitochondria were expressed relative to the myofibrillar space and SS mitochondria were expressed relative to the fiber surface area. The total volumetric content of mitochondria (IMF + SS) was calculated as IMF + SS/20 based on the assumption that the fibers were of cylindrical shape with a diameter of 80 μm and that the volume beneath the surface area was equal to a triangular prism with the dimensions of 1 × 1 × 40 µm. The analyses of volumetric content were conducted by two investigators. An assessment of inter-investigator reproducibility indicated a coefficient of variation of 6% and a bias of 14%. The raw data for mitochondrial volume fractions were adjusted for this bias. The precision of the estimates was evaluated as proposed by [25] for stereological ratio estimates. This revealed an estimated coefficient of error (CE) of 0.07 for IMF mitochondria per muscle and 0.14 for SS mitochondria per muscle.

The mitochondrial cristae surface area per mitochondrial volume (SV) was estimated by the equation, SV = 2 IL/Rm, where IL indicates intersections per test lines and Rm is a correction factor for unidentified inner membranes [26]. Grid sizes of 90 and 270 nm were used to estimate mitochondria volume and cristae surface area, respectively. Rm was estimated by a test line system counting intersections with the outer membrane, where the double-leaflet structure is apparent compared to the total trace of the outer membrane (Figure 2D). Then, it was assumed that the same fraction of unidentified membranes also applied to the membranes of the cristae. Mitochondrial profiles were included randomly, but only profiles with a Rm ≥ 1/3 were included in the analyses. One blinded investigator conducted all the cristae analyses. We analyzed a high number (median of 61 and range of 18–131) of mitochondrial profiles for cristae surface area density, which corresponds to an estimated CE of 0.09 per muscle. In the analyses of fiber-type differences, fewer muscle fibers and, hence, mitochondrial profiles could be included (see fiber typing above). Therefore, we plotted the estimated CE against the number mitochondrial profiles analyzed (Figure 2C) and decided that at least 12 mitochondrial profiles should be analyzed to achieve a satisfactorily high precision.

### 2.8. Immunoblotting

Frozen muscle tissue was freeze dried, homogenized, resolved by sodium dodecyl sulfate polyacrylamide gel electrophoresis, and electroblotted onto polyvinylidene difluoride membranes, as previously described [27]. Membranes were incubated overnight at 4 °C in primary antibodies in a 1:1000 TBST solution. The following commercially available primary antibodies were purchased from Abcam: dynamin-related protein-1 (DRP1) (#ab56788), mitofusin-1 (MFN1) (ab57602), optic atrophy-1 (OPA1) (#ab4236), CS (#ab96600), 3-hydroxyacyl-CoA dehydrogenase (β-HAD) (#ab81492), Parkin (#ab15954), PINK1 (#ab75487), Beclin1 (#ab55878). The following commercially available primary antibodies were purchased from Cell Signaling Technology: COXIV (#4844), light chain 3 beta (LC3B) (#3868), UNC-51-like kinase 1 (ULK1) (#8054). All primary antibodies were used in a 1:1000 TBST solution with 5% BSA. After incubation in primary antibodies, membranes were incubated at room temperature in horseradish peroxidase-conjugated secondary antibodies for the appropriate host species in a 1:5000 TBST solution with 1% BSA for 1 h. The proteins of interest were visualized with a chemiluminescent substrate (Thermo Scientific, Waltham, MA, USA). Arbitrary protein intensity was quantified with a UVP imaging system (UVP, Upland, CA, USA) and normalized to total protein loaded in the corresponding lane using Stain-Free technology as previously described [28,29]. A representative stain-free blot image used for the normalization of total protein is shown in supplementary materials, Figure S1.

### 2.9. Statistics

All statistical analyses were performed with STATA 15.0 (StataCorp, College Station, TX, USA). Anthropometric and clinical characteristics of CLI and CON patients were compared using unpaired t-tests for continuous variables and the chi-square test for categorical variables. Similarly, differences in mitochondrial outcomes between CLI and CON patients were analyzed using unpaired t-tests. Fiber-type-specific differences in mitochondrial content and cristae density were analyzed using a linear mixed model. Variables were analyzed with subject-ID as a random effect and fiber type and group as fixed effects. Model validation included tests for equal standard deviations and examination of QQ-plots. Correlations between mitochondrial volumetric content and markers of mitochondrial content were investigated by calculation of Pearson’s correlation coefficient and Lin’s coefficient of concordance (Rc). Lin’s coefficient of concordance is defined on a scale from 0 to 1 where 0.21–0.4 indicates a fair concordance, 0.41–0.60 a moderate concordance, 0.61–0.80 a substantial concordance, and 0.8–1.0 a nearly perfect concordance [30]. Similar to the study by Larsen et al., only markers that were significantly associated and had a Rc above 0.61 were considered valid biomarkers [31]. For all statistical tests, *p* < 0.05 was considered statistically significant. Data in graphs are presented as individual values with means ± SD or individual values with medians (25th–75th percentile).

## 3. Results

### 3.1. Anthropometric and Clinical Characteristics

Anthropometric and clinical characteristics are presented in Table 1. We found no differences between groups with respect to age, sex, weight, height, BMI, hypertension, diabetes mellitus, lung disease, hypercholesterolemia, nephropathy, or use of ACE-inhibitors, metformin, diuretics, statins, antibiotics, and insulin. CLI patients had a lower ABI compared to CON (*p* < 0.01) and a higher occurrence of related ischemic symptoms (rest pain (*p* < 0.01), wounds (*p* < 0.01), and gangrene (*p* < 0.01)). CLI patients were more frequent smokers than CON (*p* < 0.01).

### 3.2. Global Mitochondrial Respiration

**Figure 3:**
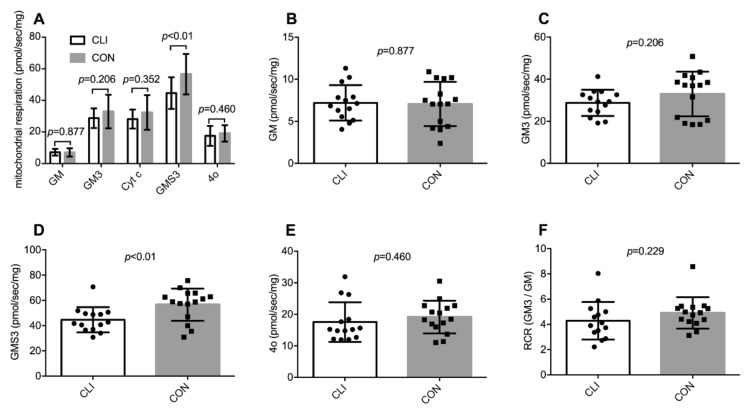
Mitochondrial respiration per mg muscle tissue for CLI (n = 14) and CON (n = 15) patients. (**A**) Summary of all respiratory states after addition of: glutamate and malate (GM), ADP (GM3), cytochrome c (cyt c), succinate (GMS3), and oligomycin (4o). (**B**–**F**) Individual values for all respiratory states and the respiratory control ratio (RCR) with complex I-linked substrates. Data are presented as means ± SD.

Mitochondrial respiration per mg wet weight of muscle tissue is shown in Figure 3. State 3 respiration supported by glutamate/malate/succinate was 12.0 (95% CI: 3.2,20.8) pmol/mg/s (21%) lower in CLI patients (44.6 ± 9.9 pmol/mg/s) than CON (56.6 ± 12.8 pmol/mg/s) (*p* < 0.01). For all other respiratory states, we did not detect any statistical differences between groups. Two fiber bundles were excluded based on the cytochrome c test. In these cases, data are based on one fiber bundle. In the cytochrome c-test negative fiber bundles, the addition of cytochrome c did not increase respiration (−0.6% ± 4.8%), suggesting that the isolation and permeabilization procedures did no damage to the outer mitochondrial membrane.

### 3.3. Mitochondrial Volumetric Content

**Figure 4:**
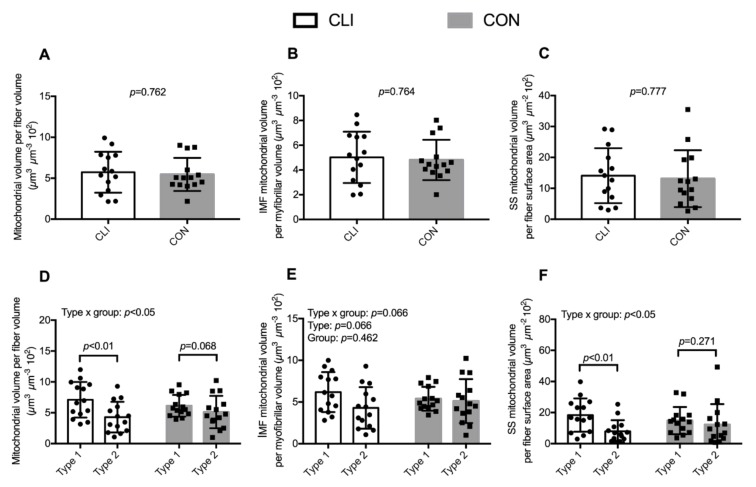
Mitochondrial content for CLI (n = 14) and CON (n = 14) patients. (**A**–**C**) Total, IMF, and SS mitochondrial volume fraction, respectively. (**D**–**F**) Total, IMF, and SS mitochondrial volume fraction stratified for fiber type, respectively. Data are presented as means ± SD. Overall linear mixed model effects are designated in the upper left corner of graphs D–F.

Total and subpopulation mitochondrial volumetric content for whole muscle and stratified by fiber type are presented in Figure 4. At the whole-muscle level, we did not detect any differences between CLI patients and CON with regard to total mitochondrial volume fraction, IMF mitochondrial volume fraction, and SS mitochondrial volume per fiber surface area. Among the two subpopulations, IMF mitochondria were quantitatively the largest contributing with 89% ± 3% to total mitochondria for CLI and 89% ± 4% for CON (data not shown).

There was a fiber type x group interaction for total mitochondrial volume fraction (*p* < 0.05). The total mitochondrial volume fraction was 2.81 (95% CI: 1.3,4.3) µm^3^ µm^−3^ 10^2^ (40%) lower in type 2 fibers (4.3 ± 2.5 µm^−3^ 10^2^) than type 1 fibers (7.1 ± 2.9 µm^−3^ 10^2^) for CLI patients (*p* < 0.01). There was a fiber type x group interaction for SS mitochondrial volume per fiber surface area (*p* < 0.05). The SS mitochondrial volume per fiber surface area was 10.4 (95% CI: 4.8,16.1) µm^3^ µm^−2^ 10^2^ (56%) lower in type 2 fibers (8.0 ± 7 µm^3^ µm^−2^ 10^2^) than type 1 fibers (18.4 ± 10.7 µm^3^ µm^−2^ 10^2^) for CLI patients (*p* < 0.01).

### 3.4. Common Markers of Mitochondrial Content

**Figure 5:**
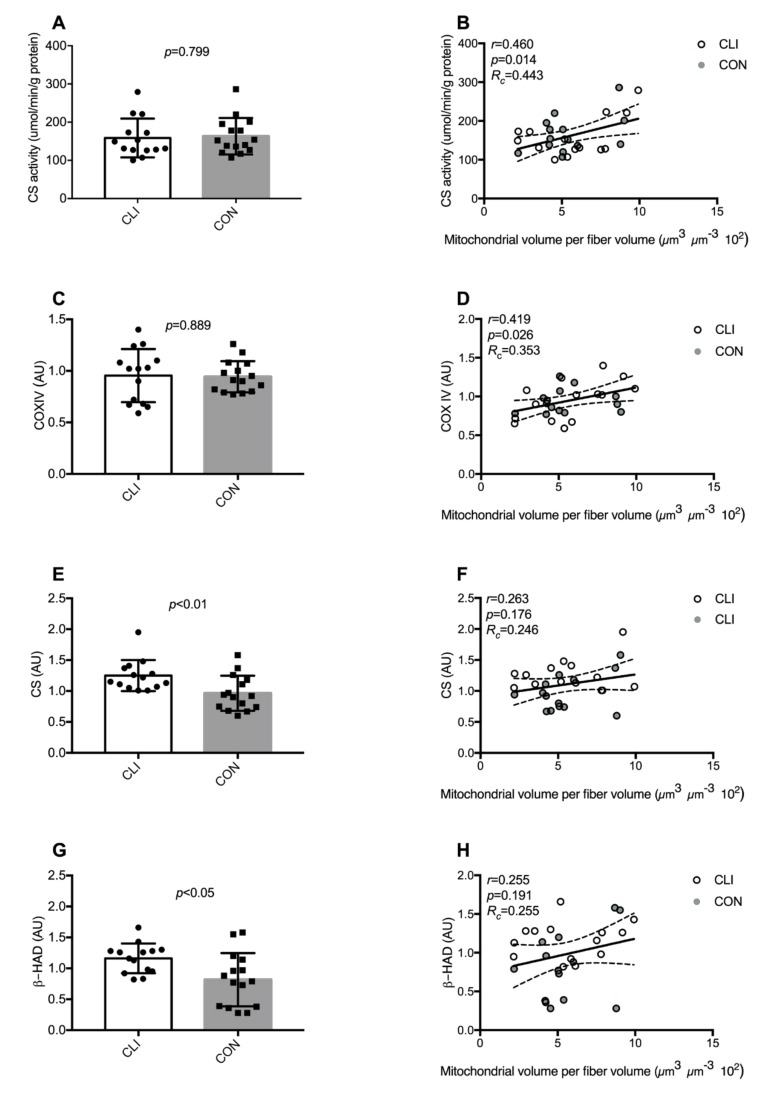
Markers of mitochondrial content. (**A**) Citrate synthase (CS) activity, (**C**) expression of cytochrome c oxidase subunit IV (COXIV), (**E**) CS, and (**G**) 3-hydroxyacyl-CoA dehydrogenase (β-HAD) for CLI (n = 14) and CON (n = 15). Data are presented as means ± SD. Association between (**B**) CS activity, (**D**) COXIV protein expression, (**F**) CS protein expression, (**H**) β-HAD protein expression and mitochondrial volumetric content for all patients pooled (CLI (n = 14) and CON (n = 14)). Data are presented as individual values with line of fit (95% CI). Pearson’s correlation coefficient (r), significance (p), and Lin’s coefficient of concordance (*R*_c_) are designated in the upper left corners of the graphs.

Markers commonly used to predict mitochondrial content are presented in Figure 5. β-HAD (*p* < 0.05) and CS protein expression (*p* < 0.01) were higher in CLI patients compared to CON. Similar differences were not detected for CS-activity and COXIV protein expression. Only CS activity (*p* < 0.05) and COXIV protein expression (*p* < 0.05) were significantly associated to mitochondrial content, but Lin’s test of concordance revealed that CS activity (*R*_c_ = 0.46) and COXIV protein expression (*R*_c_ = 0.35) only exhibited a fair-to-moderate concordance with mitochondrial volumetric content with both groups pooled. Stratification for the groups did not change the interpretation of the results and, hence, only the pooled analyses are shown in graphs.

### 3.5. Cristae Content

**Figure 6:**
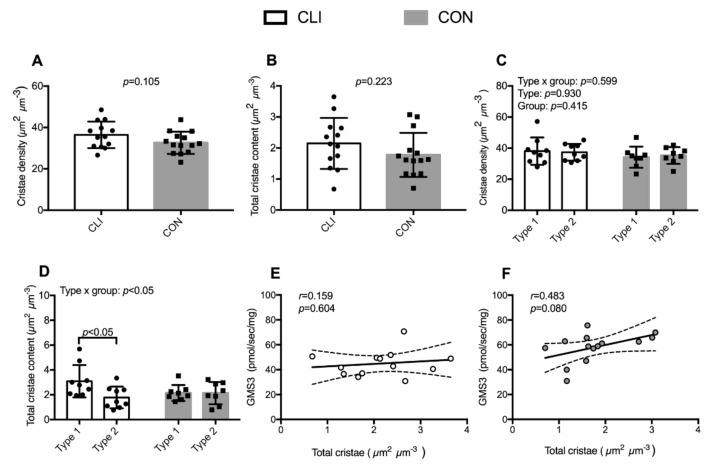
Whole-muscle (CLI: n = 13, CON: n = 14) and fiber-type-specific (CLI: n = 9, CON: n = 8) cristae density and content. (**A**) Whole-muscle cristae density, (**B**) fiber-type-specific cristae density, (**C**) whole-muscle total cristae content, and (**D**) fiber-type-specific total cristae content. Data are presented as means ± SD. Overall linear mixed model effects are designated in the upper left corners of graphs B and D. Association of whole-muscle total cristae content and GMS3 respiration for (**E**) CLI patients and (**F**) CON. Data are presented as individual values with line of fit (95% CI). Pearson’s correlation coefficient (*r*) and significance (p) are designated in upper left corners of the graphs.

Whole-muscle and fiber-type-specific cristae density and content are presented in Figure 6. At the whole-muscle level, we did not detect any statistical differences between groups with regard to cristae density and total cristae content. When stratifying for fiber type, there was a fiber type x group interaction for total cristae content (*p* < 0.05). Total cristae content was 1.3 (95% CI: 0.5,2.2) µm^2^ µm^−3^ (42%) lower in type 2 fibers (1.8 ± 0.9 µm^2^ µm^−3^) than type 1 fibers (3.1 ± 1.3 µm^2^ µm^−3^) for CLI (*p* < 0.05). There was a borderline positive correlation between total cristae content and state 3 respiration supported by glutamate/malate/succinate for CON (*r* = 0.483, *p* = 0.080). A similar relationship was not detected for CLI patients (*r* = 0.151, *p* = 0.604).

### 3.6. Mitochondrial Content-Specific Respiration and Cristae-Specific Respiration

**Figure 7:**
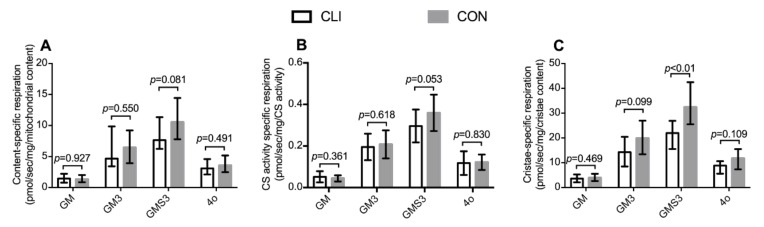
Mitochondrial respiration per mitochondrial volumetric content, CS activity, and cristae content. (**A**) Mitochondrial volumetric content-specific respiration; CLI (n = 14) and CON (n = 14) patients, (**B**) CS activity-specific respiration; CLI (n = 14) and CON (n = 15), and (**C**) cristae-specific respiration; CLI (n = 13) and CON (n = 14). Respiratory states after addition of glutamate and malate (GM), ADP (GM3), succinate (GMS3), and oligomycin (4o). Data are presented as (B) means ± SD and (A, C) medians (25th−75th) percentile.

Mitochondrial volumetric content-specific, CS activity-specific, and cristae-specific respiration are presented in Figure 7. Normalization of the respirometric results to mitochondrial volumetric content, citrate synthase activity, and total cristae surface area did not change the overall pattern between groups. For specific *p*-values, see the respective figures.

### 3.7. Expression of Mitophagy-Related and Fission/Fusion Proteins

**Figure 8:**
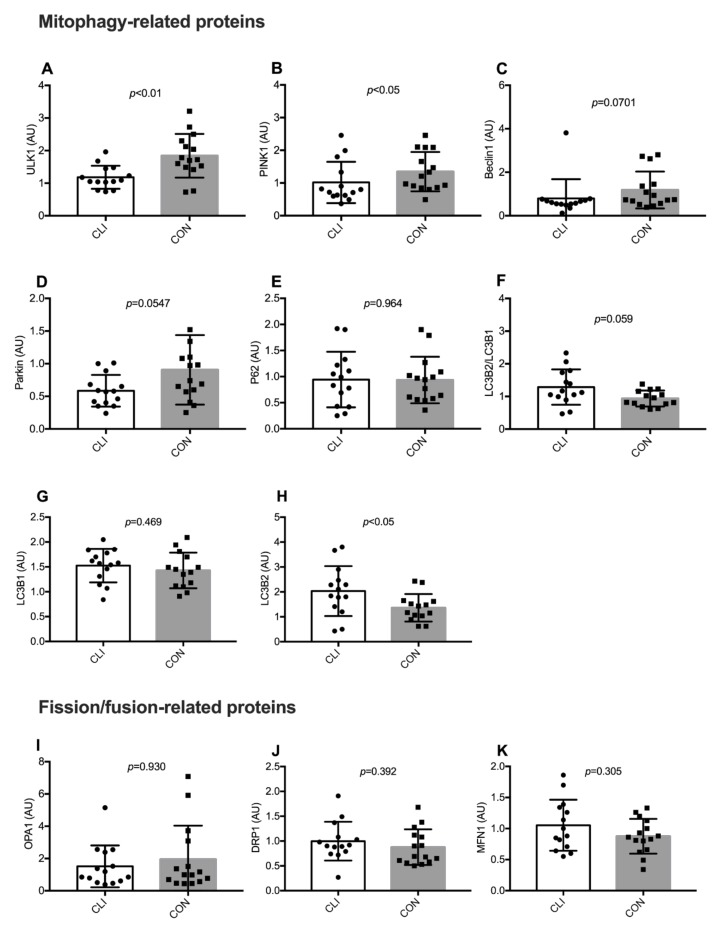
Expression of mitophagy and fission/fusion-related proteins for CLI (n = 14) and CON (n = 15) patients. (**A**) UNC-51-like kinase 1 (ULK1), (**B**) PTEN-induced kinase 1 (PINK1), (**C**) Beclin, (D) Parkin, (**E**) sequestosome-1 (P62), (**F**) ratio between microtubule-associated protein 1 light chain 3 beta (LC3B) 2 and 1, (**G**) LC3B1, (**H**) LC3B2, (**I**) optic atrophy-1 (OPA1), (**J**) dynamin-related protein-1 (DRP1), (**K**) Mitofusin-1 (MFN1). Data are presented as means ± SD (A, E, G, H, J, K) and medians (25th−75th) percentile (B, C, D, F, I).

Expression of mitophagy and fission/fusion-related proteins are shown in Figure 8. Expression of ULK1 (*p* < 0.01) and PINK1 (*p* < 0.05) were lower in CLI patients than CON. Expression of Parkin (*p* < 0.054) and Beclin1 (*p* = 0.070) were borderline lower in CLI patients than CON. LC3B2 was higher (*p* < 0.05) and the LC3B2/LC3B1 ratio (*p* = 0.059) was borderline higher in CLI patients compared to CON. Similar differences between groups were not detected for P62 and LC3B1. We did not detect any differences between groups for expression of the fission/fusion-related proteins OPA1, DRP1, or MFN1. Representative blot images of individual proteins and a representative stain-free blot image used for normalization of the total protein are shown in the supplementary material Figure S1.

## 4. Discussion

The main finding of the current study is that the ischemic skeletal muscle of CLI patients exhibit a distinctive impairment in mitochondrial respiration when compared to a closely matched CON group with similar medication regime and comorbidity risk profile. Unlike other conditions characterized by mitochondrial dysfunction, the respiratory deficit in CLI patients is independent from altered mitochondrial content or cristae density. Our results suggest that this intrinsic mitochondrial respiratory deficit in CLI patients is linked to dysregulation of PINK1-Parkin-mediated mitophagy, which is a central pathway involved in mitochondrial quality control. These results may have implications for future development of therapeutic strategies targeting the myopathy accompanying CLI patients.

In the present study, we show that the ischemic skeletal muscle of CLI patients is characterized by reduced mitochondrial respiration supported by glutamate/malate/succinate indicating impaired ATP production capacity. This is in accordance with previous observational and preclinical studies using respirometry to assess mitochondrial function in different models of CLI [12,13,15,16]. We have been able to establish a unique CON group with a similar medication regime and general comorbidity risk profile, and our results indeed support a contention that mitochondrial dysfunction is a hallmark of CLI, which could represent a therapeutic target in combatting the myopathy associated with CLI. To clarify whether mitochondrial dysfunction in CLI patients is associated with altered mitochondrial content and/or intrinsic changes in the mitochondrial reticulum, we performed an exhaustive characterization of whole-muscle and fiber-type-specific mitochondrial volumetric content and cristae density. By this approach, no differences were observed in whole-muscle total, IMF, or SS mitochondrial volumetric content between CLI patients and CON. To our knowledge, this is the first study to assess mitochondrial content with transmission electron microscopy in CLI patients. Based on assessments of CS activity and cardiolipin content, which has previously been validated as biomarkers of mitochondrial content in healthy individuals [31], a recent report suggested that CLI patients have maintained mitochondrial content compared to PAD patients with less severe manifestations and healthy controls [15]. While our results confirm that mitochondrial content is maintained in CLI patients, we also show that the validity of citrate synthase, COXIV, and β-HAD cannot reliably be extrapolated for use in CLI patients or in patients with ischemic heart disease. Considering the widely accepted use of citrate synthase activity as a proxy for mitochondrial content, these results call for concern on the use of this marker in all patient populations where the validity has not yet been established.

Whereas we found no difference in mitochondrial volumetric content between CLI patients and CON, previous studies assessing mitochondrial volumetric content in PAD patients with intermittent claudication have suggested that mitochondria accumulate in the ischemic muscle tissue [32,33]. The discrepant findings may support the concept that muscle tissue from patients with intermittent claudication and CLI harbor different mitochondrial phenotypes [15].

Our observations of inferior mitochondrial respiration and maintained mitochondrial content strongly suggest that the mitochondrial reticulum is dysfunctional in CLI patients. Yet, we did not detect any differences in cristae density between the two patient groups. If anything, cristae density was higher in CLI patients mediating an even larger mitochondrial respiratory deficit when respiration was normalized to cristae content. To our knowledge, this is the first study to assess mitochondrial cristae density in CLI patients and the first study in general to provide information on respiration per cristae content in humans. In healthy young individuals, it was previously reported that total cristae content correlated with maximal coupled respiration [31]. In the current study, a similar relationship was observed for CON but not CLI patients. The observed dissociation between mitochondrial respiration and cristae content in CLI patients represents a novel characteristic of CLI patients and invites similar studies in other patients characterized by mitochondrial dysfunction (e.g., COPD and diabetes patients). Future studies should also investigate other intrinsic mitochondrial properties such as supercomplex formation [34] and post-translational modifications [35] to elucidate mechanisms for intrinsic mitochondrial dysfunction in CLI patients.

Whereas we did not detect any differences between CLI patients and CON with respect to whole-muscle mitochondrial and cristae content, stratification of fiber types revealed different patterns. Overall, mitochondria were more abundant in type 1 fibers than in type 2 fibers, but the difference in abundance between type 1 and type 2 fibers was more pronounced for CLI than CON. A similar pattern was observed specifically in the IMF and SS mitochondrial populations. Previous studies suggest that the degree of myofiber denervation and oxidative damage in PAD patients is most severe in type 2 fibers and parallels disease severity [36,37]. The greater difference in mitochondrial content between fiber types in CLI patients may reflect that type 1 and 2 fibers respond differently to an ischemic environment, emphasizing the importance of future investigations of fiber-type-specific alterations in CLI patients.

To explore whether mitochondrial dysfunction in CLI patients is linked to altered mitochondrial quality control, we looked at the protein expression of proteins involved in PINK1-Parkin-mediated mitophagy and fission/fusion-related processes. We show that several proteins involved in PINK1-Parkin-mediated mitophagy exhibit altered expression in CLI patients with lower ULK1 and PINK1 expression and a trend toward lower Beclin1 and Parkin expression. When mitochondria lose their membrane potential or increase production of reactive oxygen species, PINK1 is recruited to the outer mitochondrial membrane where it recruits and activates the E3 ligase Parkin [38]. More recently, UNC-51-like kinase 1 (ULK1)-mediated phosphorylation of Beclin1 was also shown to facilitate translocation of Parkin to the outer mitochondrial membrane [39]. Parkin can poly-ubiquitinate proteins on the outer mitochondrial membrane [40], which leads to binding of adaptor proteins such as P62 that can, in turn, bind microtubule-associated protein 1A/1B-lightchain 3 (LC3)-II, leading to mitophagy [38,41]. Consequently, our results may indicate impaired capacity for initiation of PINK1-Parkin-mediated mitophagy and, hence, dysregulation of mitochondrial quality control. Our results do not firmly appoint impaired mitochondrial quality control and PINK1-Parkin-mediated mitophagy as a cause for mitochondrial dysfunction in CLI patients, but emphasize it as a potential attack point for future experimental studies and impending therapies.

As mitochondrial content was preserved in CLI patients, our findings indicate that other degradation pathways compensate for the impaired PINK1/Parkin-mediated mitophagy or that mitochondrial biogenesis is decreased so that the net turnover of mitochondrial reticular components is balanced. In contrast to other proteins involved in PINK1-Parkin-mediated mitophagy, LC3B-II content and the LC3BII/LC3BI ratio were higher in CLI patients compared to CON, indicating either increased activation of autophagy or decreased clearance of autophagosomes [42]. In addition, it needs to be emphasized that LC3B and ULK1 are not specific to PINK1-Parkin-mediated mitophagy but is rather a general autophagy precursor. No differences between CLI patients and CON emerged for the expression of proteins involved in fission/fusion events.

In the current study, muscle tissue was harvested during primary surgery from CLI and CON patients, who underwent general anesthesia. General anesthetics have been reported to inhibit mitochondrial respiratory function in both animal and human models [43,44]. The numeric data on mitochondrial respiration from the current study can, therefore, not be directly compared to studies using local anesthesia.

We included patients with coronary artery disease undergoing coronary artery bypass graft surgery as CON because comorbidities and risk factors for coronary artery disease resemble those of PAD [1]. Furthermore, collection of the saphenous vein during coronary artery bypass graft surgery means that no undue invasive procedures were required for the sampling of gastrocnemius muscle tissue, which further advocated for these patients as an appropriate CON in our study design. However, it should be noted that CLI patients were more frequent smokers. Differences in smoking status may act as a confounder for the observed differences in mitochondrial characteristics between PAD and CON patients [45]. Physical activity level constitutes another important determinant of skeletal muscle mitochondrial content and functionality [46], but habitual physical activity level was not evaluated. Furthermore, additional inclusion of a healthy age-matched control group could have provided information on the absolute severity of the mitochondrial abnormalities. However, the results obtained from a healthy age-matched control group would be confounded by the lack of comorbidities, medication, and sedentary lifestyle. Furthermore, muscle tissue was harvested under general anesthesia that, for ethical reasons, would have been impossible in healthy individuals.

Our results show that several biomarkers of mitochondrial content may not be considered valid in a diseased population. However, the biomarkers investigated in this study were biased by selection. As such, cardiolipin, which has previously been shown to exhibit strong association with mitochondrial content [31], was not investigated in the current study and should be given the benefit of the doubt before completely rejecting biomarkers in general as a proxy measure of mitochondrial content in patients with ischemic heart disease and CLI or patients in general.

## 5. Conclusions

CLI patients are characterized by impaired mitochondrial respiration in the ischemic muscle, which is not explained by altered mitochondrial volumetric content or cristae density. The dissociation between mitochondrial function and structure represent a novel characteristic of CLI patients. Our results suggest that impaired mitochondrial function in CLI patients is linked to dysregulation of the mitochondrial quality control system PINK1-Parkin-mediated mitophagy, which may be of importance for future development of novel therapeutic strategies targeting the myopathy associated with CLI. Finally, we show that three commonly used biomarkers of mitochondrial content cannot be considered valid in patients with ischemic heart disease or CLI. These results call for concern on the use of these biomarkers in general diseased populations where their validity has not been confirmed.

## Figures and Tables

**Table 1:** Anthropometric and clinical characteristics.

	CON (n = 15)	CLI (n = 14)	*p*
Age (y)	67.4 ± 7.4	65.3 ± 7.8	0.463
Sex (male/female)	15/0	13/1	0.292
Height (cm)	178.2 ± 8.1	178.0 ± 8.4	0.948
Weight (kg)	89.5 ± 15.8	89.8 ± 17.7	0.973
BMI (kg/m^2^)	28.0 ± 3.3	28.1 ± 4.3	0.958
ABI	1.11 ± 0.13	0.41 ± 0.19	<0.01
Lower leg rest pain (Y/N)	0/15	7/7	<0.01
Ischemic wounds (Y/N)	0/15	6/8	<0.01
Gangrene (Y/N)	0/15	6/8	<0.01
Smoking (current/past/non)	1/5/9	10/4/0	<0.01
Hypertension (Y/N)	5/10	6/8	0.597
Diabetes mellitus (Y/N)	4/11	5/9	0.599
Lung disease (Y/N)	2/13	5/9	0.159
Nephropathy (Y/N)	0/15	1/13	0.292
Hypercholesterolemia (Y/N)	3/12	3/11	0.924
Statins (Y/N)	15/0	11/3	0.058
Metformin (Y/N)	4/11	3/11	0.742
Diuretics (Y/N)	7/8	6/8	0.837
Antibiotics (Y/N)	11/4	8/6	0.359
ACE-inhibitors (Y/N)	5/10	4/10	0.782
Insulin (Y/N)	4/11	4/10	0.909

Continuous data are shown as means ± SD. Categorical data are shown as number of subjects.

## Supplementary Materials

The following are available online at https://www.mdpi.com/2073-4409/9/3/570/s1, Figure S1: Representative immunoblots.
