# Supplementary material for: Mitochondrial Structure and Function in the Metabolic Myopathy Accompanying Patients with Critical Limb Ischemia
Source: Cells. 2020 Feb 28;9(3):570. doi: 10.3390/cells9030570 (PMC7140415; doi:10.3390/cells9030570)
Supplement: Supplementary file 1 [file cells-09-00570-s001.pdf]

SUPPLEMENTAL MATERIAL

Mitochondrial structure and function in the metabolic myopathy accompanying patients with critical limb ischemia

Thomas Groennebaek[1], Tine Borum Billeskov[2,6], Camilla Tvede Schytz[1,3], Nichlas Riise Jespersen[4], Hans Erik Bøtker[4], Rikke Kathrine Jentoft Olsen[5], Nikolaj Eldrup[6], Joachim Nielsen[3], Jean Farup[2], Frank Vincenzo de Paoli[2,6*], Kristian Vissing[1*]

[1]Department of Public Health, Aarhus University, Aarhus, Denmark
[2]Department of Biomedicine, Aarhus University, Aarhus, Denmark
[3]Department of Sports Science and Clinical Biomechanics, University of Southern Denmark, Odense, Denmark
[4]Department of Cardiology, Aarhus University Hospital, Aarhus, Denmark
[5]Research Unit for Molecular Medicine, Aarhus University Hospital, Aarhus, Denmark
[6]Department of Cardiothoracic and Vascular Surgery, Aarhus University Hospital, Aarhus, Denmark

Supplemental figure

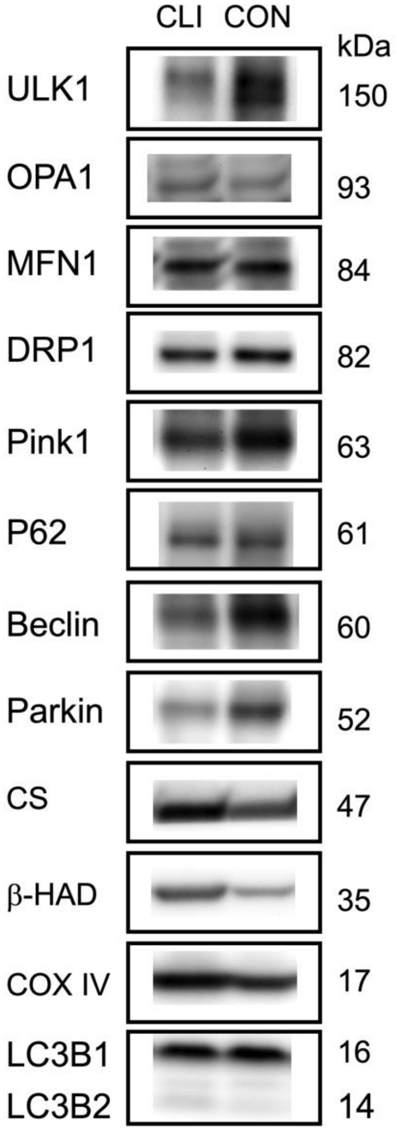

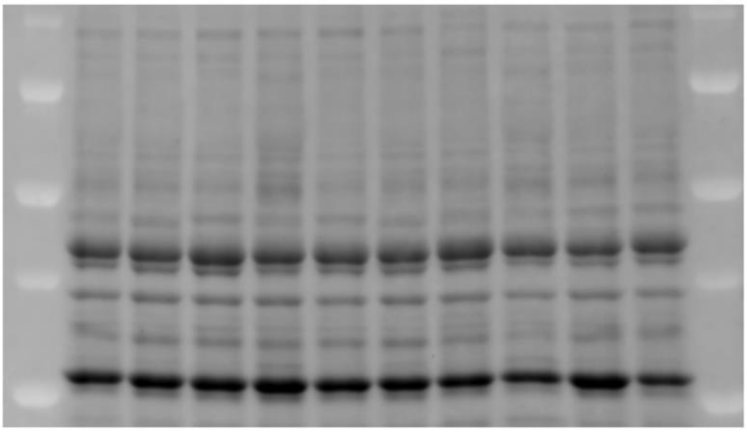

Figure S1. Representative immunoblots. Representative blots of individual proteins for one CLI patient and one CON patient(**A**). Representative stain free blot image of membrane used for normalization of total protein for five CLI patients and five CON patients (**B**).
